# Zn-Based Alloys for Plain Bearings—Influence of Al and Cu Content on Mechanical Properties

**DOI:** 10.3390/ma17051062

**Published:** 2024-02-26

**Authors:** Angelika Kiefel, Steffen Gimmler, Christoph Broeckmann, Uwe Vroomen

**Affiliations:** 1 Institute for Materials Applications in Mechanical Engineering, 52062 Aachen, Germany; c.broeckmann@iwm.rwth-aachen.de; 2 Foundry Institute, RWTH Aachen University, 52072 Aachen, Germany; s.gimmler@gi.rwth-aachen.de (S.G.); u.vroomen@gi.rwth-aachen.de (U.V.)

**Keywords:** hardness, compression test, tensile test, fracture analysis, tribological test

## Abstract

In recent decades, the requirements for plain bearing materials have continually increased, especially with new applications such as wind turbines, which require larger bearings. These new applications have completely different property profiles compared with, for example, bearings in automotive construction. Larger bearings need high strength and wear resistance, which established bearing materials cannot fulfill. Therefore, new alloy systems are required. This publication focuses on the influence of alloy composition and test temperature on the mechanical properties of ZnAlCu alloys. Centrifugally cast specimens were produced for the fabrication of test specimens, which were used to determine the mechanical and tribological properties. Fracture surface and wear trace analysis with scanning electron and light microscopy were used to determine occurring failure and wear mechanisms and to analyze the influence of microstructure on failure. Depending on the composition of the ZnAlCu alloys, up to three times higher strengths can be achieved compared with the white metal alloy SnSb12Cu6ZnAg. Furthermore, all the alloys investigated show good wear properties. Up to 11 wt.% aluminum and 1.5 wt.% copper, a significant decrease in the wear coefficient was observed. Knowledge about the correlation between microstructure, properties, and failure mechanisms of ZnAlCu alloys can be used to produce bearing metal alloys suitable for a wide range of applications. Since the strength values lie between those of white metals and bronze, new fields of application can also be accessed.

## 1. Introduction

Currently, Zn-based alloys are mainly used as non-structural components such as locking systems, safety components, handles, and fittings [1,2]. These alloys also show high strength and hardness, as well as good corrosion and wear resistance, especially in combination with Al and Cu. These properties make them interesting as plain-bearing materials [3,4,5,6,7]. Existing studies of alloys of the ternary system (zinc, aluminum, and copper) have already shown that these alloys can certainly meet the requirements for plain bearings. Especially in the area of eutectoid composition, there are already various studies that substantiate this potential [8,9]. However, the requirements for plain-bearing materials are high, often conflicting, and constantly increasing. Typical requirements are good emergency running properties combined with high mechanical strength and high wear resistance. In addition, economic requirements are also increasing because of rising raw material prices and increasing health and environmental awareness [10,11,12]. Plain-bearing materials, such as white metals, can no longer fulfill these mechanical and economic requirements. This paper focuses on the mechanical and tribological properties of hypoeutectoid ZnAlCu alloys as a function of their alloy composition and the test temperature. Both tensile and compression tests are considered. On the one hand, tensile tests are better suited to evaluate the ductility of materials. On the other hand, plain bearings are mainly subjected to compressive loads, which have so far been insufficiently investigated in the literature, with a lack of results considering hypoeutectic ZnAlCu alloys. The obtained results are compared with those of the white metal alloy SnSb12Cu6ZnAg.

## 2. Materials and Methods

Samples with compositions ranging from 1 wt.% to 20 wt.% aluminum and from 0.1 wt.% to 3 wt.% copper were produced by melting pure zinc (Zn 99.995%), pure aluminum (99.8%), and pure copper (99.99%) in an induction furnace. The resulting homogenized melt was first cast into a steel die. Then the melt was preheated by 150 °C and subsequently cooled at a constant rate of about 0.8 °C/s.

For the mechanical characterization, hardness measurements, as well as tensile and compression tests, were carried out. According to DIN ISO 4384-2 [13] for hardness tests of bearing metals, the Brinell hardness test was performed. A testing machine of the Otto Wolpert, type DiaTestor 3a, was used. All tests were carried out at a stress level of HB10 and the test condition of HBW2.5/62.5/10. 

A universal testing machine from ZwickRoell type Z020 (Ulm, Germany) with a maximum force application of 20 kN and a quartz glass extensometer were used for tensile and compression tests. Tensile tests were performed on round specimens with a test diameter of 5 mm and a parallel test length of 30 mm. Compression tests, on the other hand, were performed on cylindrical test specimens with a height and diameter of 7 mm. Fractographic analysis of the fracture surfaces of the tensile samples was performed by scanning electron microscopy (SEM) with a type Jeol JSM-6400 microscope (Freising, Germany).

Specimens for the tribological tests were cut and ground up to 1000 grit to minimize the influence of surface roughness on friction and wear properties. The tribological tests were carried out on a microtribometer (Anton Paar, Graz, Austria).

The fracture surfaces and wear traces of tested samples were examined by SEM (Zeiss, Oberkochen, Germany) and energy dispersive X-ray spectroscopy (EDS) (Oxford Instruments, Wiesbaden, Germany) to analyze the morphology and composition.

## 3. Results and Discussion

### 3.1. Microstructure

**Figure 1:**
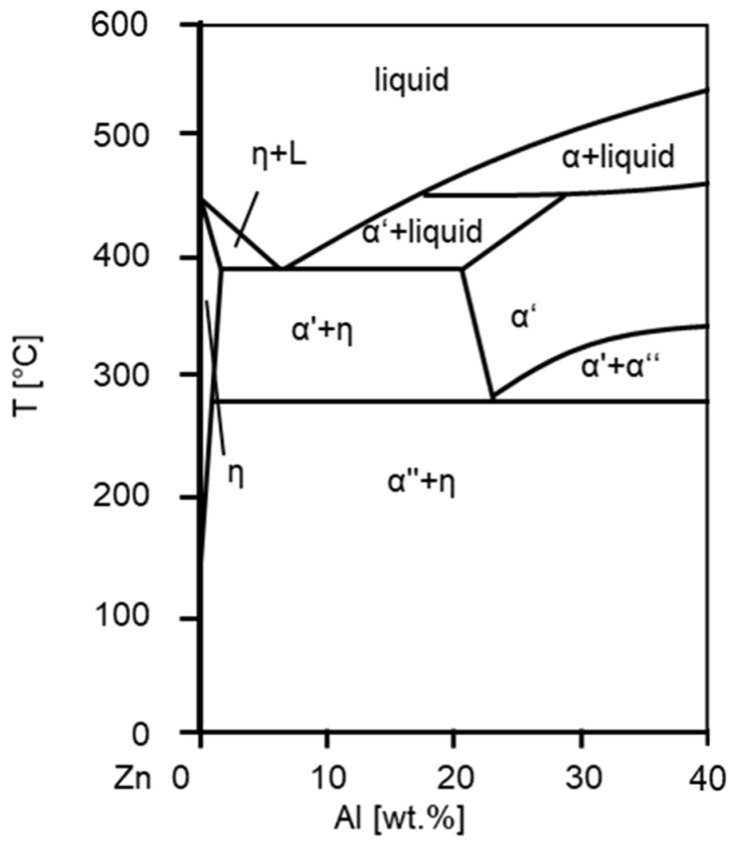
Zn-Al phase diagram.

**Figure 2:**
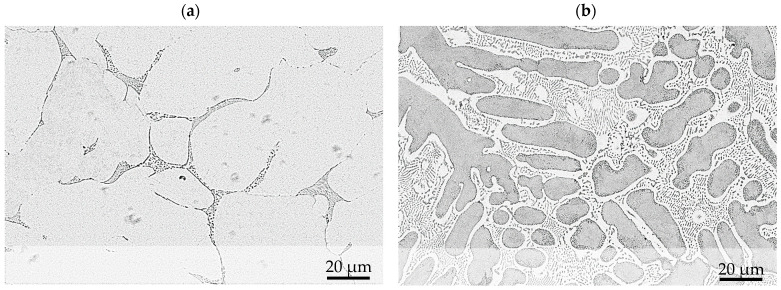
Microstructure of the hypoeutectic alloy ZnAl1Cu0.7 (**a**) and the hypereutectic alloy ZnAl11Cu0.7 (**b**).

The strength and wear resistance of ZnAlCu alloys are determined by the content of Al and Cu and are closely linked to the solidification process. Figure 1 shows the binary Zn-Al phase diagram. Considering near-equilibrium solidification in hypoeutectic alloys with an aluminum content of less than 5 wt.%, the zinc phase η is first precipitated from the melt. At 382 °C, the residual melt then eutectically decomposes into η and the zinc-rich aluminum phase α′. The solubility of aluminum in the η-phase decreases with cooling until the zinc-rich α′ decomposes at 275 °C through a eutectoid reaction into the η phase and the zinc-poor aluminum phase α″. At the end of the solidification process, the microstructure is characterized by a combination of the primarily solidified η phase and a fine lamellar eutectic and eutectoid structure. Hypereutectic alloys solidify initially in the aluminum-rich α-phase. Then they decompose in the same eutectic and eutectoid reaction as hypoeutectic alloys, resulting in a similar microstructure with fine eutectic and eutectoid lamellae but in an initially solidified α-phase. In the ternary system ZnAlCu, the intermetallic ε-phase with the composition Zn4Cu is formed. Up to 2 wt.% Cu, this leads to an increase in hardness and strength. Figure 2 shows the microstructure of a hypoeutectic and hypereutectic alloy [14,15,16,17].

### 3.2. Hardness

The Brinell hardness test was performed on the alloys ZnAl4Cu0.7, ZnAl5.5Cu0.7, ZnAl5.5Cu3, ZnAl11Cu0.7, and ZnAl20Cu0.7. The results obtained are listed in Table 1. The hardness of all alloys considered is between 75 HB10 and 112 HB10. Comparing the alloys with aluminum contents of more than 4 wt.% and copper contents of more than 0.7 wt.% with the alloy ZnAl4Cu0.7, an increase in hardness with increasing alloy content can be observed. Only the eutectic alloy ZnAl5.5Cu0.7 has a lower hardness than ZnAl4Cu0.7, despite the higher alloy content. 

In order to use ZnAlCu alloys for plain bearing metals, the hardness of the ZnAlCu alloy in correlation to the shaft hardness must not exceed a certain hardness value in order to exclude damage to the shaft. This hardness value is based on empirical values and is interpreted differently in the literature. In one approach proposed by K. Droste, it is assumed that the hardness difference between the bearing and shaft must be at least 100 HB [18]. This leads to maximum allowable hardnesses of the bearing metal of 107 HB30 for a shaft made of C45 or to maximum allowable hardness values of 141 HB30 for a shaft made of 42CrMo4. In another approach, it is assumed that there must be a minimum factor of three between the hardness values of the two sliding partners [19]. This results in significantly lower maximum hardness values of 69 HB30 for a C45 shaft and 80 HB30 for a 42CrMo4 shaft compared with the first approach. A third approach leads to similar maximum hardnesses for the bearing metal as the second approach. It is assumed that the ratio of hardness and Young’s modulus of the two sliding partners must differ by a factor of at least 1.5 [20,21].

In order to compare hardness values according to Brinell, all tests must be carried out under the same stress level, irrespective of the selected test condition. The necessary stress level for different materials is listed in DIN EN ISO 6506-1:2015-02 [22] and is determined by the evaluability of the indentation diameter. 

According to DIN EN ISO 6506-1:2015-02 [22], the condition for an evaluable indentation diameter is as shown in Equation (1).

0.24D < d < 0.6D(1)

with D = ball diameter and d = indentation diameter.

For the evaluation of the three approaches the ZnAlCu alloys have to be compared with the shaft material. Since the shaft material has a significantly higher strength than ZnAlCu alloys, the hardness test of the shaft material must be carried out with a stress level of HB30. The hardness test of alloys similar to Zn-based alloys, on the other hand, must be carried out with a stress level of HB10 [13,22].

The bearing metal SnSb12Cu6ZnAg used as a reference alloy is significantly softer than the ZnAlCu alloys and, according to DIN ISO 4384-2:2014-07 [13], has to be tested with a stress level of HB2.5. Consequently, the hardness values are only comparable to a limited extent.

If the Brinell hardnesses are nevertheless compared with each other, the Brinell hardness of SnSb12Cu6ZnAg with 24HB is significantly below the Brinell hardness of all ZnAlCu alloys examined [23]. Compared to C45 and 42CrMo4, the ZnAlCu alloys only fulfill the approach according to K. Droste [18].

### 3.3. Compression Test

Investigations of material behavior under uniaxial compressive loading were carried out at room temperature (RT), which means at a temperature of about 25 °C, and 100 °C. A 3-zone furnace from MTS type 652.01D was used for the tests at 100 °C. The traverse speed of the crosshead was 0.014 mm/s for all tests.

In order to prevent bending or buckling during the test according to DIN 50106 [24], a ratio of the initial height *h*_0_ to the initial diameter *d*_0_ according to equation 2 was chosen.

(2) $$1\leq \frac{h_{0}}{d_{0}}\leq 2$$

Datasheets frequently give the 0.2% and 2% compression yield strength as well as the compression strength as characteristic values for material behavior under uniaxial compressive loading. The specification of the compressive strength is defined in DIN 50106 [24] and requires a specimen fracture. 

**Figure 3:**
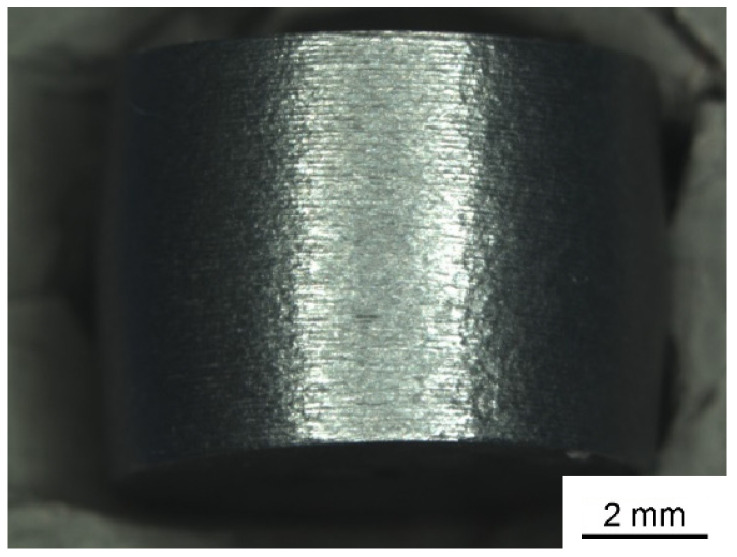
ZnAl20Cu0.7 pressure sample after the compression test.

Figure 3 shows a compression sample of the alloy ZnAl20Cu0.7 tested by RT. There is a slight bulge occurring during the compression test but no fracture or even significant cracks at the surface of the sample. Low alloyed samples with about 4 wt.% aluminum show first cracks in the surface. However, since no specimen fracture occurred in the investigated Zn alloys the 0.2% compression yield strength was selected as the characteristic value for characterization. 

**Figure 4:**
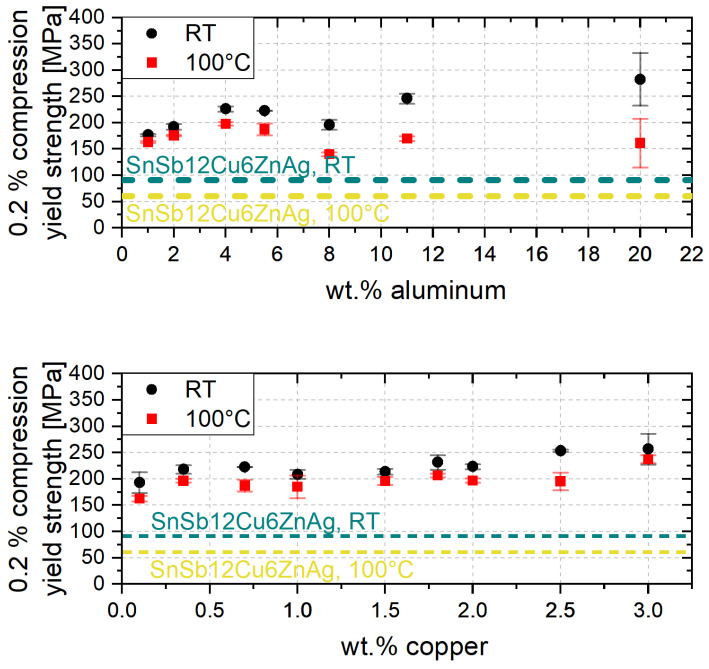
A 0.2% compression yield strength over aluminum and copper content.

The dependence of aluminum content at 0.7 wt.% copper and copper content at 5.5 wt.% aluminum is shown in Figure 4 at RT and 100 °C. Independent from the testing temperature a slight increase of the 0.2% compression yield strength can be observed with increasing aluminum and copper content. The tests at 100 °C show a lower 0.2% compression yield strength for all alloy compositions compared to 0.2% compression yield strength at RT. Furthermore, at low aluminum contents, the 0.2% compression yield strength at RT and 100 °C differ only slightly from each other, whereas the temperature influence increases with higher aluminum content. At an aluminum content of 20 wt.% the 0.2% compression yield strength at RT is about 120 MPa higher than at 100 °C. 

The increase of the 0.2% compression yield strength with higher aluminum and copper content can be attributed to solid solution strengthening [25]. Since the aluminum-rich solid solution strengthened α phase has a significantly higher strength than the zinc-rich η-phase, an increasing α-phase content results in an increase in strength. Below 2 wt.% copper the increase in strength is also due to a significant increase in solid solution strengthening. From a copper content of 2 wt.%, on the other hand, hard intermetallic Zn4Cu precipitates form which leads to an increase in strength [1,26]. Furthermore, the lower 0.2% compression yield strength at 100 °C is caused by the thermal energy input. At high temperatures, dislocations can overcome obstacles more easily, so that at 100 °C the 0.2% compression yield strength is already reached at lower stresses than at RT. Since a higher proportion of eutectic and eutectoid structures is present with increasing aluminum content, the influence of the energy input is greater than with low aluminum contents. Therefore, the difference in the 0.2% compression yield strength at RT and 100 °C is greater at higher aluminum contents.

In comparison to the white metal alloy SnSb12Cu6ZnAg often used in plain bearings the 0.2% compression yield strength is at least twice as high for all zinc-base alloys investigated at RT and 100 °C. The higher yield strength can lead back to the higher strength of the zinc-rich η matrix compared to the tin matrix in SnSb12Cu6ZnAg.

### 3.4. Tensile Tests

**Figure 5:**
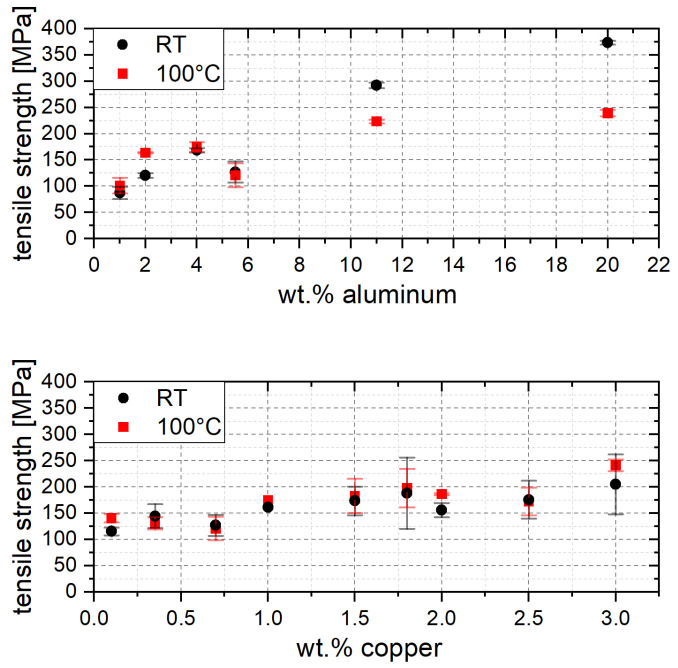
Tensile strength in dependence of the aluminum and copper content.

The tensile strength behaves equivalently to the 0.2% compression yield strength of the ZnAlCu alloys. With increasing aluminum and copper content, an increase in tensile strength is observed. Furthermore, a greater temperature influence can also be observed with increasing aluminum content. Figure 5 shows the influence of the alloy content on tensile strength.

**Figure 6:**
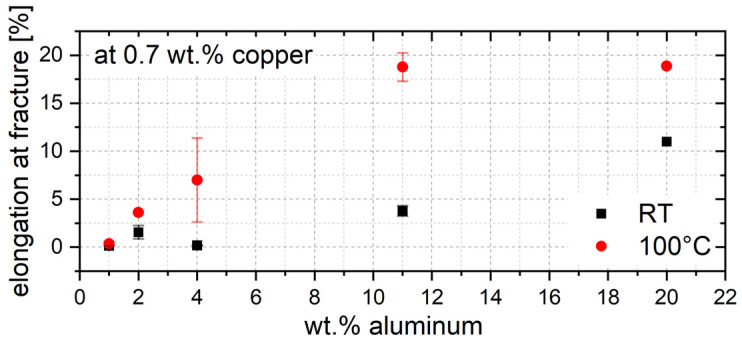
Elongation at fracture in dependence of aluminum content.

Unlike compression tests, tensile tests offer the possibility to quantitatively evaluate the ductility of a material. Figure 6 shows the influence of the aluminum content on the ductility at RT and 100 °C. The most striking feature is the very brittle material behavior of the ZnAlCu alloys with a low aluminum content. Since the copper content in all the alloys investigated is between 0.1 wt.% and 3 wt.%, which is also very low, the material is almost pure zinc when the aluminum content is low. Due to the hexagonal lattice structure of zinc, spontaneous sliding occurs along the base of the hexagonal lattice structure, resulting in sudden failure with little or no plastic deformation. On the other hand, with increasing aluminum content, the proportion of aluminum-rich α-phase increases. The face-centered cubic lattice structure has more slip planes than the hexagonal lattice structure, which leads to an increased elongation. 

**Figure 7:**
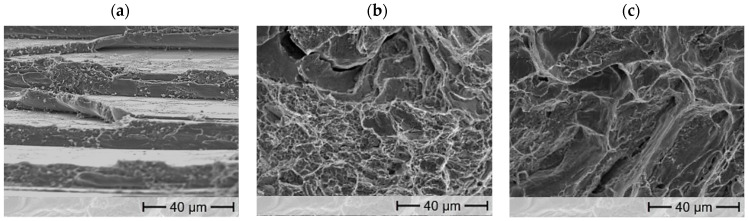
Fracture surface of (**a**) ZnAl4Cu0.7 (RT), (**b**) ZnAl20Cu0.7 (RT), and (**c**) ZnAl4Cu0.7 (100 °C).

The fracture surfaces in Figure 7 clearly show these differences using the example of ZnAl4Cu0.7 for low Al contents and ZnAl20Cu0.7 for high Al contents. The fracture surface of ZnAl4Cu0.7, Figure 7a), at RT shows a high crystallinity, whereas the fracture surface of ZnAl20Cu0.7, Figure 7b), appears much duller, which is a sign of higher ductility associated with α-phase [27]. The SEM images show the cleavage fracture typical of brittle materials in the alloy ZnAl4Cu0.7. In contrast, both cleavage planes and honeycombs are visible in the alloy with 20 wt.% aluminum, indicating rosette fracture [28].

Higher temperatures cause an additional increase in ductility, since due to thermally activated processes increased dislocation movements take place, which lead to macroscopic plastic deformation. Especially in the alloys with low aluminum contents, this difference is clearly visible in the fracture pattern. As shown in Figure 7c), the fracture surface is duller than in the room temperature tests with small honeycombs on the cleavage planes, which usually result from local constrictions.

The influence of the copper content on the elongation at fracture is very small in the investigated range and it can be assumed that the results are within the scatter band due to the small number of samples of three per alloy. To complete the results, the values determined for the elongation at fracture are listed in Table 2.

### 3.5. Tribological Tests

Tribological studies were carried out using a microtribometer to determine the influences of aluminum and copper contents on the frictional and wear properties of ZnAlCu alloys. All measurements were performed in a linear reciprocal setup without additional lubricants. The counterparts and measurement parameters used in the investigations are shown below in Table 3. The load of 1 N was chosen to surpass the compressive strength of all tested alloys with the initial pressure, equally. This ensures the comparability of the measurement of all alloys tested.

Using the Hertzian relationships described in Equations (3) and (4) and assuming a uniform elastic modulus of 96 GPa for all ZnAlCu alloys studied here, the maximum contact pressure can be estimated to be about 480 MPa.

(3) $$P0=\sqrt[{3}]{\frac{6F_{N}E^{*2}}{\pi^{3}R^{2}}}$$

(4) $$\frac{1}{E^{*}}=\frac{1-\nu_{1}^{2}}{E_{1}}+\frac{1-\nu_{2}^{2}}{E_{2}}$$

In the previous equations, P0 describes the maximum pressure in the contact zone, FN is the acting normal force, and E* is the reduced modulus of elasticity, which is calculated as described in Equation (4).

#### 3.5.1. Friction

**Figure 8:**
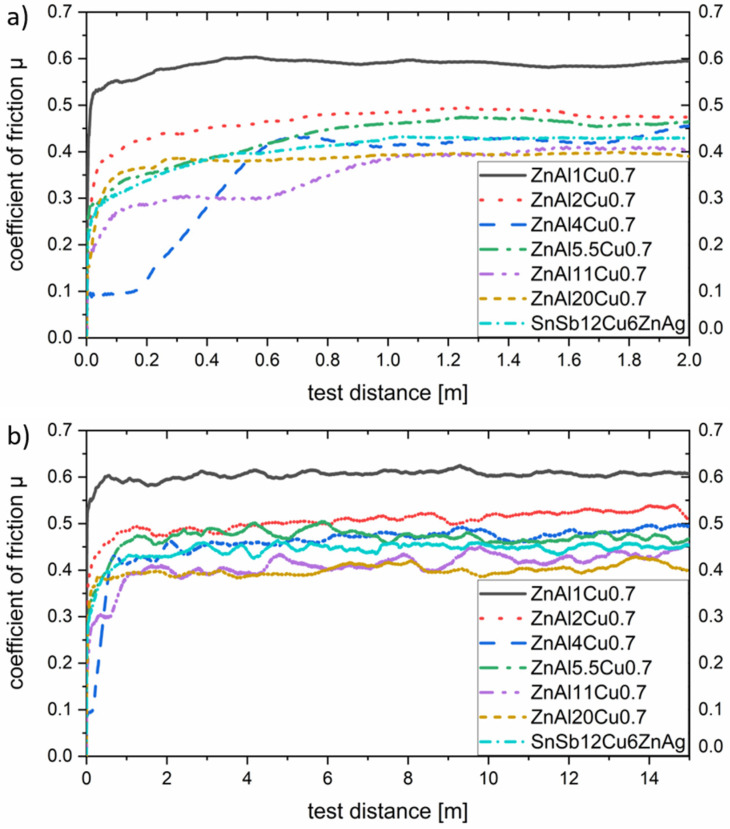
Coefficient of friction for a variation of aluminum concentrations. Part (**a**) shows the run-in area in detail and (**b**) the change in the coefficient of friction over the whole test distance.

To determine the coefficient of friction, the mean value of three measurements was calculated in each case. All measurements were carried out in the load spectrum already described. The influence of the Al content in the range from 1 wt.% to 20 wt.% on the coefficient of friction for a constant Cu concentration of 0.7 wt.% is shown below in Figure 8.

As shown in Figure 8a, there is no clear correlation between the aluminum content and the associated proportion of α-phase and the tribological behavior in the run-in area. This circumstance can be attributed to the presence of oxide layers and adhesion films on the surfaces of the tested samples. The preparation process before tribological testing can lead to the formation of oxide or hydroxide layers, which determine the tribological behavior until they slide off. The breaking through and detachment of these layers is accompanied by a significant increase in the coefficient of friction as shown above. This behavior can especially be detected for alloy ZnAl4Cu0.7, where the run-in process takes a test distance of about 0.8 m. The decrease in the coefficient of friction due to the geometric adaptation of the sliding partners, which is typical for metallic materials, is completely absent in all the specimens investigated.

All samples tested reached a steady state after approximately 1.6 m. Subsequently, the coefficient of friction remained almost constant. In this range, a clear tendency of the aluminum content on the resulting friction can be seen. An increase in the Al content thus leads to a reduction in friction. This can be attributed to the hardness, which also increases with increasing α-phase content, but also to the changed wear behavior discussed later. In the load spectrum investigated, a coefficient of friction of about 0.4 could be achieved from an Al content of 11 wt.%. Higher contents did not show any significant improvement in the frictional properties.

**Figure 9:**
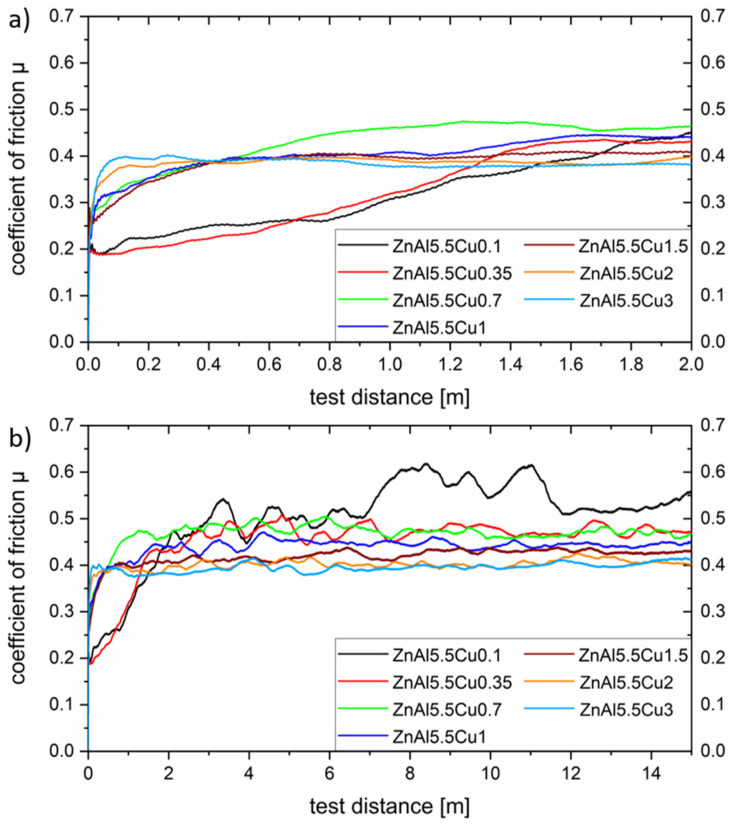
Coefficient of friction for a variation of copper concentrations and constant aluminum concentration. Part (**a**) shows the run-in area in detail and (**b**) the change in the coefficient of friction over the whole test distance.

In agreement with Jareño et al. [29], initial studies of phase formation in alloys in the ZnAlCu system have shown that the two intermetallic phases ε (Zn4Cu) and T’ (Al5Cu4Zn) increasingly form above a copper content of 2 wt.%. Therefore, the investigations of the tribological properties are limited to the range of up to 3 wt.% Cu in order to avoid a too-pronounced formation of these two hard phases. According to Savaşkan et al. [26] these two hard phases act as an abrasive third phase in the tribological contact. Results of the tribological measurements for various alloys with different copper contents are shown in Figure 9.

Unlike varying the aluminum content, increasing the copper content shows a significant effect on the running-in behavior under tribological loading. As shown in Figure 9a, an increase in the Cu content leads to a shortening of the running-in time. This can be mainly attributed to the reduced formation of stable hydroxide layers during sample preparation. The running-in time for low copper contents, on the other hand, is significantly increased by about 3.2 m.

When looking at the coefficient of friction in the steady state in Figure 9b, it becomes clear that here, too, the addition of copper lowers this coefficient decisively. This effect is most pronounced up to copper contents of 1.5 wt.%, and becomes almost negligible beyond that, which is consistent with the saturation limit for solid solution strengthening. These observations are in accordance with the results of Savaşkan et al. where a decrease in the coefficient of friction was observed up to a copper content of 2 wt.%. Spatially resolved phase field simulations have shown that at a Cu content of 3 wt.%, there is an ε-phase content of about 14%. Under the shown test conditions this does not seem to have a significant effect on the friction coefficient yet. The intermediate jump in the coefficient of friction when testing the sample with the composition ZnAl5.5Cu0.1 between 8 m and 12 m may have been caused both by the presence of microporosity in the test specimen or by the break-out of a coarse wear particle.

#### 3.5.2. Wear

**Figure 10:**
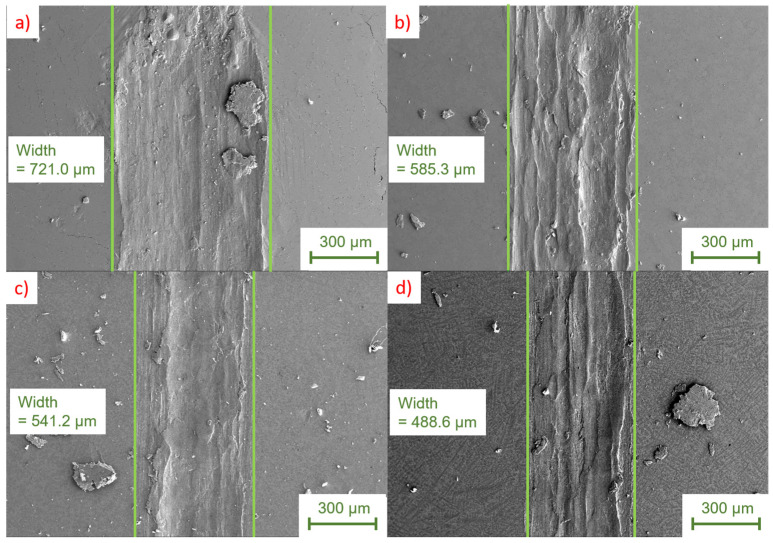
Comparison of wear track widths for different aluminum concentrations after tribological loading in the described load spectrum. (**a**) ZnAl1Cu0.7; (**b**) ZnAl4Cu0.7; (**c**) ZnAl11Cu0.7; (**d**) ZnAl20Cu0.7. All the images show a secondary electron contrast generated at an accelerating voltage of 20 KV.

To quantify the wear of the investigated tribological tracks, the width of the generated wear tracks is used for a first estimation. This wear track width was determined on the recorded SEM images. Together with the EDS measurements carried out in this context, it provides information about the prevailing tribological processes and tribochemical phase transformations. A comparison of the track widths on samples with different aluminum concentrations is given below in Figure 10.

**Figure 11:**
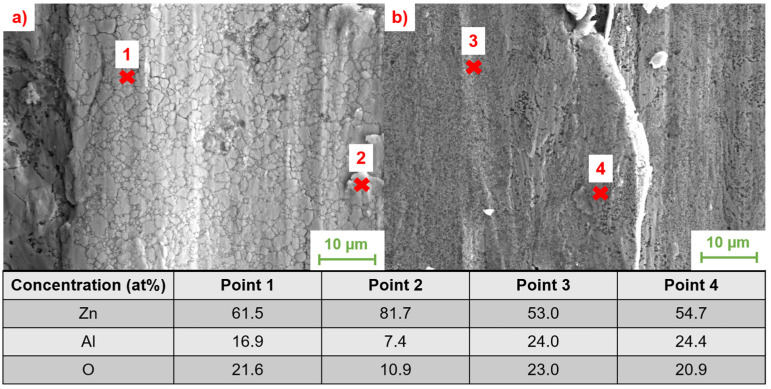
SEM images with a magnification of 5000× of (**a**) ZnAl1Cu0.7 and (**b**) ZnAl11Cu0.7. The composition of the points measured with EDS is listed in the table.

From the images shown, it is clear that an increase in Al content and the associated increase in α-phase leads to a significant reduction in wear track widths. This increase in wear resistance can be attributed on the one hand to the compressive strength and hardness, which are also increased with a higher α-phase content, but also to a possible change in the wear mechanisms. In order to investigate the wear mechanisms in more detail and to detect tribochemical transformations in the contact zone, EDS measurements were performed at higher magnifications on wear particles and in the wear track. A comparison of the wear traces with the locations used for the EDS measurements, as well as the measured composition in tabular form, is shown below in Figure 11.

As can be seen from the EDS measurements carried out, a reaction layer of aluminum oxide is formed in both alloys under the induced tribological load. However, this layer is much more pronounced and stable for the alloy ZnAl11Cu0.7. For alloys with lower Al content, the layer also appears to have a strong tendency to fragment under cyclic tribological loading due to the lower strength of the matrix. These fragments of the alumina layer, in contrast to the solid layer at higher Al concentrations, do not show a high wear resistance, which also explains the increased material removal in the conducted investigations.

**Figure 12:**
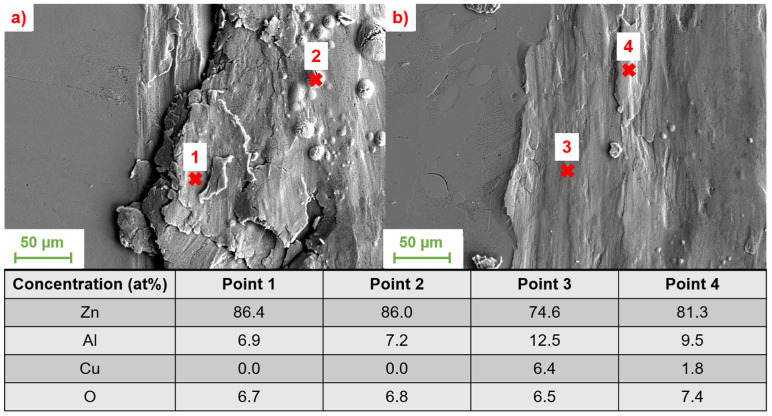
SEM images with a magnification of 1000× of (**a**) ZnAl5.5Cu0.1 and (**b**) ZnAl5.5Cu3. The composition of the points measured with EDS is listed in the table.

In addition to the effects of the copper content on the coefficient of friction already discussed, the influence of the copper content on wear was also investigated. Here, too, only copper contents up to 3 wt.% were considered in order to keep the formation of the intermetallic ε-phase as low as possible. The results of the EDS measurements for the samples with minimum and maximum concentrations of copper are shown together with the SEM images in Figure 12.

**Figure 13:**
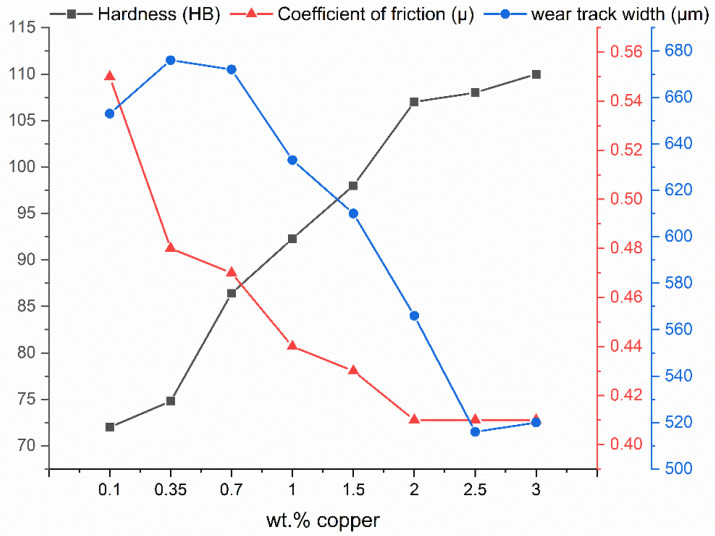
Dependence of Brinell hardness, coefficient of friction and wear track width on copper content X for a base alloy ZnAl5.5CuX.

Increasing the copper content had no effect on the composition of the tribochemically formed layer in the samples studied. As can be seen from the EDS measurements shown, with both 0.1 wt.% Cu and 3 wt.% Cu, the formation of an alumina layer under the dry friction present is the dominant mechanism. The formation of intermetallic ε or T’ phases could not be observed under the present tribological loading. Due to the fact that there are no differences in the tribochemical formation of the phases in the contact zone, the increase in wear resistance can be attributed to the higher compressive strength and hardness of the alloy by increasing the amount of solid solution hardening with higher copper content. The dependencies of the friction and wear properties, as well as the hardness, on the copper content of the alloys are shown below in Figure 13.

The dependency of the friction coefficient and the wear track width can generally be described as a linear function of the copper content up to a copper content of 2 wt.%. Above this point, a further increase in copper content causes no change in the coefficient of friction while the wear seems to slightly increase.

## 4. Conclusions

Hardness measurements, compression, and tensile tests as well as tribological tests were carried out to characterize hypoeutectoid ZnAlCu alloys in comparison to the white metal alloy SnSb12Cu6ZnAg often used as plain bearing material. The results of the work can be summarized as follows:

- The Brinell hardness measurements of the investigated alloys show hardness values between 75 HB and 112 HB. An increase in hardness can be observed with increasing alloy content. The only exception is the eutectic alloy ZnAl5.5Cu0.7, which with 75HB has a lower hardness than ZnAl4Cu0.7 with 84HB. Compared to the white metal alloy SnSb12Cu6ZnAg, however, the hardness of all alloys investigated is at least three times as high.
- The evaluation of the compression tests shows a slight increase in the 0.2% compression yield strength with increasing Al and Cu content due to solid solution strengthening and the formation of the hard intermetallic Zn4Cu precipitation at copper contents above 2 wt.%. The measured 0.2% compression yield strength at 100 °C is lower than at RT with increasing differences at higher aluminum contents. This can be attributed to the thermal energy input, which leads to increased dislocation sliding. Compared to the white metal alloy SnSb12Cu6ZnAg, the investigated alloy system shows up to 300% higher 0.2% compression yield strength.
- Equivalently to the 0.2% compression yield strength the tensile strength increases with higher Al and Cu content. The elongation at fracture of hypoeutectic alloy compositions at RT of max. 0.74% is clearly below the elongation at fracture of SnSb12Cu6ZnAg. Hypereutectic compositions, on the other hand, not only show a high increase in strength but are also characterized by an elongation at fracture that is approximately four times as high. Considering other research [27,30], according to which the elongation at break decreases with increasing aluminum content, this is a surprising result that requires further investigation. Tests at 100 °C show a decrease in strength with a simultaneous increase in elongation at fracture compared to tests at RT.
- The coefficient of friction becomes smaller with increasing aluminum and copper content which can be attributed to the higher compression strength and hardness. Furthermore, a comparison of the alloy ZnAl20Cu0.7 with SnSb12Cu6ZnAg within the considered load spectrum showed a 25% higher wear resistance.

Considering the described results ZnAlCu alloys with Al and Cu contents up to 20 wt.% Al and 3 wt.% Cu shows higher mechanical and better tribological properties than SnSb12Cu6ZnAg. However, to fully evaluate hypoeutectoid alloys in the context of plain bearings further investigations e.g., on fatigue behavior, are necessary. 

## Figures and Tables

**Table 1:** Results of the hardness test under the test condition of HBW2.5/62.5/10.

Stress Level	ZnAl4Cu0.7	ZnAl5.5Cu0.7	ZnAl5.5Cu3	ZnAl11Cu0.7	ZnAl20Cu0.7
HB10	84	75	110	104	112

**Table 2:** Elongation at fracture at RT and 100 °C.

Alloy	Elongation at Fracture at RT [%]	Elongation at Fracture at 100 °C [%]
SnSb12Cu6ZnAg	2.91	8.62
ZnAl1Cu0.7	0.14	0.42
ZnAl2Cu0.7	0.37	3.58
ZnAl4Cu0.7	0.17	6.95
ZnAl5.5Cu0.1	0.17	3.16
ZnAl5.5Cu0.35	0.11	0.84
ZnAl5.5Cu0.7	0.49	2.50
ZnAl5.5Cu1	0.26	1.10
ZnAl5.5Cu1.5	0.24	4.37
ZnAl5.5Cu1.8	0.64	3.09
ZnAl5.5Cu2	0.26	1.08
ZnAl5.5Cu2.5	0.26	2.38
ZnAl5.5Cu3	0.74	3.21
ZnAl11Cu0.7	3.77	18.70
ZnAl11Cu2	5.32	20.97
ZnAl20Cu0.7	11.05	20.72

**Table 3:** Settings for the tribological investigations.

Counterpart		Loading	Speed	Track Lenght	Cycles	Temperature	Humidity
Material	Diameter						
100Cr6	6 mm	1 N	5 cm/s	10 mm	800	23 °C	45 %

## Data Availability

Data are contained within the article.
